# Sleep or Play Online Poker?: Gambling Behaviors and Tilt Symptoms While Sleep Deprived

**DOI:** 10.3389/fpsyt.2020.600092

**Published:** 2021-01-11

**Authors:** Alexandre Hamel, Célyne Bastien, Christian Jacques, Axelle Moreau, Isabelle Giroux

**Affiliations:** ^1^Centre québécois d'excellence pour la prévention et le traitement du jeu (CQEPTJ), École de psychologie, Université Laval, Québec, QC, Canada; ^2^Laboratoire de neurosciences humaines comportementales – Sommeil et Potentiels Évoqués (SPEC), École de psychologie, Université Laval, Québec, QC, Canada; ^3^Centre intégré universitaire de santé et de services sociaux du Centre-Sud-de-l'Île-de-Montréal, Institut universitaire sur les dépendances, Montréal, QC, Canada

**Keywords:** online gambling behavior, tilt, sleep deprivation, poker, sleep quality

## Abstract

Online poker has the convenience of being accessible 24/7 allowing a large proportion of players to gamble at night. Although some studies postulate a bi-directional relationship between excessive online poker playing and sleep disturbances, sleep has yet to be studied as a primary outcome variable in online poker studies. Sleep deprivation has been linked to alterations in emotional regulation, decision-making, and risk-taking behaviors. All of which are known to induce episodes of tilt. Conversely, online poker playing during regular sleep hours may interfere with sleep quality. The objectives of the present study are (a) to explore the effects of sleep deprivation on tilt symptoms and gambling behaviors and (b) to assess whether playing an online poker session shortly before bedtime (120 min) influences the player's sleep quality. Sleeping habits, tilt symptoms, and online poker behaviors of 23 regular online poker players (22 men, 1 woman) were monitored daily for 28 days using questionnaires and hand histories. Tilt and gambling behaviors during online poker sessions (*n* = 588) played while the player was sleep-deprived were compared to sessions played while not sleep-deprived. Different sleep variables were also compared for sessions (*n* = 897) played 2 h before bedtime to no sessions played before sleep. Sleep-deprived poker sessions revealed higher emotional and behavioral tilt, a higher number of hands played and unfavorable financial results than at-rest sessions. Also, emotional and behavioral tilt was higher when alcohol was consumed. Sessions played 2 h before bedtime revealed a shorter sleep onset latency than when no sessions were played before bedtime. *Post*-*hoc* mixed regression analyses revealed that emotional and behavioral tilt is associated with shorter total sleep time and shorter sleep onset latency, while cognitive tilt is associated with a decrease in sleep efficiency. This study is the first to specifically explore sleep variables with online poker players within an ecological study design. The findings shed light on the daily impacts of nighttime online gambling practices. Future studies are needed to further explore the interaction between subjective and objective sleep variables and online gambling habits as well as investigate players' motives for playing while sleep deprived.

## Introduction

Poker is a gambling card game that has seen a significant increase in popularity since the beginning of the 2000s (1, 2). Online poker (OP), being a billion-dollar industry (3), allows players to compete with others worldwide using the electronic device of their choice at a time that is convenient for them. The 24-h accessibility of OP is a greatly appreciated characteristic of the game (4). However, this accessibility has been shown to be associated with the loss of control over gambling behavior (5). Furthermore, playing OP during regular sleep hours appears to be common and may also lead to adverse consequences for gamblers. In a survey conducted by the *Observatoire des Jeux* in France (2012; *N* = 4,042), nearly three-quarters (72.5%) of OP players reported playing late in the evening or during the night, and nearly half (45.6%) reported that OP interfered with their sleeping time (6). Late-night gambling is also possible and popular in the province of Quebec (Canada) where tournaments are offered every night via the online government website EspaceJeux.com (7).

Sleep disturbances and difficulties may affect gambling behavior. Data from the National Comorbidity Survey suggests that individuals with reported gambling problems are more likely to experience one or more sleep-related difficulties in comparison to general population (8). These sleep disturbances and difficulties may, in turn, decrease a player's ability to maintain control over their gambling behaviors and impair their decision-making ability (9, 10). Despite the research highlighting the fact that a large proportion of OP players gamble late in the evening or during the night and that sleep difficulties are associated with worrisome gambling practices, no research has specifically examined the effects of night-time gambling behaviors on the loss of control. Also, no studies have been conducted exploring the consequences of late-night gambling on sleep quality. The objectives of the present study are (a) to explore the impacts of sleep deprivation on the loss of control in OP players and (b) to explore the impacts of OP on sleep quality the night following an OP session.

Poker is a gambling card game where several players (usually 2–10) compete to win the pot. Different variations of poker exist of which Texas Hold'Em is the most popular (11, 12). Texas Hold'Em can either be played in a cash game or a tournament.

OP players are predominantly men between the ages of 26 and 35 years old (13). The reasons for playing generally include skill development, pleasure, to make money, compete but also to escape problems (14–16). OP is a gambling game with structural characteristics that differ from other forms of games of chance, such as video lottery terminals, lotteries, or scratch tickets. Unlike these pure forms of games of chance, there is a skill component present in OP that allows some more experienced and skilled players to make long-term profits (17).

Decision-making capabilities and emotional regulation are two crucial elements in OP (18, 19). Decision-making is a complex cognitive process necessary for individuals to make optimal choices according to predetermined criteria (20). Emotional regulation refers to the processes responsible for observing, evaluating, and modulating emotions, which enable an individual to accomplish goals and function in a variety of contexts (21, 22). A player willing to have an advantage in OP must be able to determine the statistics and probabilities of winning a hand based on the cards on the table and his private cards. Using this knowledge, the player must make rational choices based on the level of risk associated with each decision if he wishes to optimize the probability of long-term gains (17, 23). To do this, many techniques are employed: developing experience by playing, reading books on poker strategy, discussing poker with other players, and using tools such as hand-tracking software (24, 25). Findings by Morgan show that experienced players use probability more effectively by adjusting their level of risk-taking according to the expected winnings of a hand in comparison to less experienced players. These findings also show a relationship between negative emotions and the propensity to take risks in less experienced players. Morgan's work highlights the importance of decision-making in OP and the effect that emotions and player experiences may have on the gambler's decision-making capabilities. However, these findings stem from laboratory experiments, limiting their ecological validity. This experimental design limits the observation as to what can cause emotions and decision-making to vary leading to a loss of control over gambling behavior or the onset of tilt episodes [e.g., (26, 27)]. In poker, tilt refers to a transient loss of control of gambling behaviors associated with emotional, cognitive, and behavioral manifestations (28).

### Tilt in Online Poker

Tilt can occur because of events that may or may not be poker-related (28). For example, a tilt episode may occur following a bad hand, a loss of a large bet when the odds of winning were favorable, or as a result of intimidation by other players. Inattention, fatigue, lack of concentration, stress, drug or alcohol use may contribute to the occurrence of tilt episodes (26, 28). Emotional regulation strategies appear to be effective in preventing tilt episodes and are often used by more experienced players (26, 29). These strategies can include becoming aware of and accepting the emotions associated with the tilt episode or even momentarily leaving the game to cool down (26, 30). Tilt is known for its effects on game strategy. For example, amongst others, tilt episodes may provoke a gambler to play the game in a more aggressive manner than they would have initially (28). Tilt can lead to impaired decision-making, the illusion of control, increased risk-taking, increase the likelihood for impulsive behavior, and make it difficult for the player to stop gambling (26–29). The loss of control over gambling behaviors through tilt is associated with negative financial consequences. Indeed, tilt is believed to result in player's being less responsible when it comes to bankroll management (28, 31).

Tilt can destabilize the player and require the gambler to employ emotional regulation strategies to prevent or limit its consequences (26). A multitude of factors can cause tilt including fatigue and a lack of concentration (28). This can impair the player's ability to make optimal decisions leading to poor game sequences (26, 28). However, tilt is not the only factor that may affect the gambler during an OP game. Numerous studies have linked sleep deprivation to impaired decision-making and a reduced ability to regulate emotions (9, 22, 32–34). Sleep deprivation can be defined as an extension of an individual's wakefulness period that adversely affects their physical and psychological abilities (35). Contrary to popular belief, it is not necessary to be awake for 24 h or more to experience the negative effects of sleep deprivation. In fact, Van Dongen and colleagues (36) observed a decrease in neurobehavioral abilities after 15.84 h of wakefulness (*SD* = 0.73), although this period varies from one individual to another.

### Effects of Sleep Deprivation on Cognitive Abilities and Emotional Regulation

Sleep deprivation has been reported to be associated with impaired decision-making ability (9), increased impulsivity (37) and risk-taking when there is a chance of financial gain (32, 34). Research has shown that impaired cognitive ability increases with sleep deprivation (38). Emotional regulation has also been shown to be greatly affected by sleep deprivation. In fact, sleep deprivation has been found to have a greater effect on emotions and mood than on cognitive abilities (33). A meta-analysis exploring the effects of sleep deprivation found that participants experienced significant changes in self-reported emotions while sleep-deprived (33). Various levels of sleep deprivation are associated with a decrease in self-reported positive emotions and increase in self-reported negative emotions (39–41), as well as an alteration in the individual's ability to regulate emotions (40, 42). These emotional changes that are associated with sleep deprivation may be explained by a decrease in the threshold of emotional activation (39).

Even though research findings have shown adverse effects of sleep deprivation on critical functioning abilities (9, 32, 34, 37, 39–42), most of these effects are investigated in controlled laboratory studies providing very little ecological validity. Consequently, these results do not allow researchers to measure the impacts of sleep deprivation on participants' daily activities. Furthermore, the results highlighting the effect of sleep deprivation on risk-taking behaviors are derived from studies where the tasks are initially unknown to participants and from samples where the participants are not necessarily experienced in risk-taking activities such as poker. Considering these facts, it seems appropriate to explore how sleep deprivation affect gambling behaviors and daily functioning in OP players.

### Effects of Online Poker on Sleep

Research exploring the link between a player's sleep patterns and their ability to regulate gambling behavior is promising, yet incomplete. Indeed, as reported by Parhami et al. (8), there appears to be a bi-directional association. Problem gamblers reported poorer sleep quality and more sleep problems such as difficulty falling asleep, staying asleep, and early morning awakenings. Personal and financial consequences frequently associated with gambling problems may contribute to the reported sleep difficulties (8). In fact, symptoms such as rumination can impair sleep quality and promote long-term sleep problems (43). Internet gambling, such as OP, can also interfere with normal sleep patterns (6). Furthermore, two systematic reviews including children and adolescents found that evening use of electronic devices is associated with less total sleep time and a later bedtime (44, 45). Similarly, Higuchi et al. (46) found that participants experienced an increase in emotional activation after playing video games, resulting in a greater sleep latency. Despite these findings, no studies appear to have investigated the relationship between playing OP at night and sleep. The present study aims to compare the quality of a night's sleep when it is preceded or not by a nightly OP session.

In summary, it is possible that playing OP sessions during regular sleep hours can have various consequences. Numerous studies have associated sleep deprivation with impaired decision-making ability, increased risk-taking (9, 32, 34), alterations in emotional reactions and impaired emotional regulation (39–42). Emotional regulation and decision-making abilities are important aspects of poker and OP players' quality of play (18, 19) and altering these may favor tilt symptoms (26, 28, 29). Moreover, gambling behaviors may negatively affect sleep (8). The present study will be comparing OP sessions played in sleep deprivation (SDpr) with OP sessions played not sleep-deprived (NSDpr) on tilt symptoms and gambling behaviors. It will also compare self-reported sleep quality following sessions played within 2 h before bedtime with the absence of sessions played during this period.

### Objectives and Hypotheses

The main objective of this study is to determine whether SDpr produces a favorable context for tilt and worsen gambling behaviors among regular OP players within an ecological study design. It is expected that higher tilt scores (total score, emotional and behavioral factor and cognitive factor) and greater net losses will be observed for sessions played in SDpr compared to sessions played while NSDpr. The secondary objective is to test whether an OP session played 2 h before bedtime results in poorer sleep quality. It is expected that a later bedtime, a longer sleep-onset latency, a shorter total sleep time, a lower sleep efficiency, and a decreased feeling of rest the next day will be observed when a session is played 2 h before bedtime compared to when no session is played 2 h before bedtime.

## Materials and Methods

### Participants

Players were recruited through advertisements on forums, websites, and Facebook pages dedicated to OP. An e-mail invitation to participate in the study was also sent to Université Laval employees' and students' as well as to a list of volunteers from our center. Participants were included if they: (a) played OP at least once a week while sleep deprived (≥16 h between awakening and the end of the gambling session), (b) played OP with money on average twice a week for at least 1 month, (c) primarily played on an OP platform that allows hands to be recorded, (d) were at least 18 years of age, (e) considered themself as primarily a poker or an OP player amongst other gambling activities, (f) primarily played on a computer, and (g) agreed to monitor their sleeping and gambling habits. Participants were excluded if they were working night or rotating shift work with regular night shifts and if they devoted more than half of their playing time to gambling activities other than OP.

Thirty-five players were interested in participating in the study. Among them, two did not follow up on attempts to contact them, seven did not meet the eligibility criteria and one was excluded because of working nightshift. Of the 25 gamblers who completed the socio-demographic questionnaire, two did not provide data that would allow the research objectives to be met. Descriptive analyses were conducted on the 23 players whose responses were complete. Our participants were primarily men (95.7%), between the ages on 20 and 52 (*M* = 31.78, *SD* = 9.78) from Canada (91.3%). Twelve gamblers lost money during the data collection period (*M* = −284.70 USD; *SD* = 223.59) [−761.23, −61.10] while seven gamblers gained money (*M* = 224.18 USD; *SD* = 324.11) [13.96; 768.30]. Of the 19 players who provided their hand histories, an average of 31.32 (*SD* = 22.43) [2; 102] OP sessions and 7,456 (*SD* = 8,352.41) [1.073; 31.991] hands were played during the data collection period. Socio-demographic information, information regarding problem gambling severity and the poker experience level of participants are presented in Tables 1–3.

**Table 1:** Means, standard deviations, and demographic characteristics based on inclusion in the main analyses.

	**Players included in the analyses (*n* = 23)**	**Dropouts (*n* = 2)**
**Variables**	***M (SD)***	***M (SD)***
Age	31.8 (9.7)	32.0 (8.5)
Frequency of OP in the last 30 days	26.8 (20.9)	25.0 (7.1)
Number of hours of OP played in the last 30 days	69.2 (43.3)	87.5 (17.7)
	**Number of players (%)**	**Number of players (%)**
**Gender**		
Male	22 (95.7)	2 (100)
**Country of origin**		
Canada (Qc)	21 (91.3)	1 (50.0)
Other	2 (8.7)	1 (50.0)
**Marital status**		
Single	6 (26.1)	1 (50.0)
Common-law partner/in a relationship	15 (65.2)	1 (50.0)
Married	1 (4.3)	0 (0)
Widowed	1 (4.3)	0 (0)
**Education**		
High school	2 (8.7)	1 (50.0)
Vocational education	4 (17.4)	0 (0)
College	8 (34.8)	0 (0)
University- undergraduate	8 (34.8)	0 (0)
University- graduate	1 (4.3)	1 (50.0)
**Income**		
14 999 $ or less	4 (17.4)	0 (0)
15 000$ to 24 999$	4 (17.4)	0 (0)
25 000$ to 34 999$	2 (8.7)	0 (0)
35 000$ to 49 999$	7 (30.4)	1 (50.0)
50 000$ to 74 999$	2 (8.7)	0 (0)
75 000$ to 99 999$	1 (4.3)	0 (0)
100 000$ and +	1 (4.3)	0 (0)
**Socio-professional category**		
Full time employee	16 (69.6)	1 (50.0)
Unemployed	2 (8.7)	0 (0)
Student	5 (21.7)	1 (50.0)

**Table 2:** Distribution of players based on PGSI, PGSI-OP categories and responses to CPGI-consequences QUESTIONS.

	**Included (*n* = 23)**	**Dropouts (*n* = 2)**
**Variables**	**Number of players (%)**	**Number of players (%)**
**Problem gambling severity (PGSI)**		
Non-problem gambler	2 (8.7)	0 (0)
Low risk gambler	12 (52.2)	1 (50.0)
Moderate risk gambler	6 (26.1)	0 (0)
Problem gambler	3 (13.0)	1 (50.0)
**Problem gambling severity- Online poker (PGSI-OP)**		
Non-problem gambler	2 (8.7)	0 (0)
Low risk gambler	14 (60.9)	2 (100)
Moderate risk gambler	4 (17.4)	0 (0)
Problem gambler	3 (13.0)	0 (0)
**CPGI-Consequences 1_OP_Habits_Complicates life as a partner**		
Never	16 (69.6)	2 (100)
Sometimes	6 (26.1)	0 (0)
Most of the time	1 (4.3)	0 (0)
Almost always	0 (0)	0 (0)
**CPGI-Consequences 2_Spending less time with friends**		
Never	17 (73.9)	1 (50)
Sometimes	5 (21.7)	0 (0)
Most of the time	0 (0)	1 (50)
Almost always	1 (4.3)	0 (0)
**CPGI-Consequences 3_OP Habits Family difficulties**		
Never	22 (95.7)	2 (100)
Sometimes	1 (4.3)	0 (0)
Most of the time	0 (0)	0 (0)
Almost always	0 (0)	0 (0)
**CPGI-Consequences 4_Decreased productivity work/school**		
Never	14 (60.9)	1 (50)
Sometimes	8 (34.8)	1 (50)
Most of the time	1 (4.3)	0 (0)
Almost always	0 (0)	0 (0)
**CPGI-Consequences 5_OP Habits negative impact on neighbors**		
Never	23 (100)	2 (0)
Sometimes	0 (0)	0 (0)
Most of the time	0 (0)	0 (0)
Almost always	0 (0)	0 (0)
**CPGI-Consequences 6_Relationship problems**		
Never	10 (43.5)	1 (50)
Sometimes	10 (43.5)	1 (50)
Most of the time	3 (13.0)	0 (0)
Almost always	0 (0)	0 (0)
**CPGI-Consequences 7_Regular use of social services**		
Never	22 (95.7)	2 (100)
Sometimes	1 (4.3)	0 (0)
Most of the time	0 (0)	0 (0)
Almost always	0 (0)	0 (0)
**CPGI-Consequences 8_OP Habits Problems with friends**		
Never	22 (95.7)	1 (50)
Sometimes	1 (4.3)	1 (50)
Most of the time	0 (0)	0 (0)
Almost always	0 (0)	0 (0)
**CPGI-Consequences 9_Frequent family disagreements**		
Never	12 (52.2)	1 (50)
Sometimes	10 (43.5)	1 (50)
Most of the time	0 (0)	0 (0)
Almost always	1 (4.3)	0 (0)
**CPGI-Consequences 10_OP Habits OP co-worker consequences**		
Never	21 (91.3)	1 (50)
Sometimes	2 (8.7)	1 (50)
Most of the time	0 (0)	0 (0)
Almost always	0 (0)	0 (0)

**Table 3:** Frequency of responses to questions in the Poker Experience (PE) questionnaire.

	**Included (23 players)**	**Dropouts (2 players)**
**Variables**	**Number of players (%)**	**Number of players (%)**
**PE 1_Years of experience**		
<6 months	1 (4.3)	0 (0)
6 months to <1 year	0 (0)	0 (0)
1–5 years	4 (17.4)	0 (0)
More than 5 years	18 (78.3)	2 (100)
**PE 2_Frequency**		
Once a month or less	0 (0)	0 (0)
Every couple of weeks or so	1 (4.3)	0 (0)
Once or twice a week	9 (39.1)	0 (0)
Every day or almost everyday	13 (56.5)	2 (100)
**PE 3_Frequency of discussing theory/strategy**		
Never	2 (8.7)	0 (0)
Sometimes	8 (34.8)	1 (50)
Often	9 (39.1)	0 (0)
Almost everyday	4 (17.4)	1 (50)
**PE 4_Number of poker theory/strategy books**		
None	3 (13.0)	1 (50)
1–2	5 (21.7)	0 (0)
3–5	9 (39.1)	0 (0)
More than 5	6 (26.1)	1 (50)
**PE 5_Frequency of reading theory/strategy articles**		
Never	0 (0)	1 (50)
Sometimes	10 (43.5)	0 (0)
Often	10 (43.5)	1 (50)
Almost everyday	3 (13.0)	0 (0)
**PE 6_Level of knowledge of poker stats/odds**		
Poor	1 (4.3)	0 (0)
Average	5 (21.7)	0 (0)
Good	8 (34.8)	1 (50)
Excellent	9 (39.1)	1 (50)
**PE 7_Difficulty to calculate poker stats/odds**		
Very difficult	0 (0)	0 (0)
Somewhat difficult	2 (8.7)	1 (50)
Somewhat easy	11 (47.8)	0 (0)
Very easy	10 (43.5)	1 (50)
**PE 8_Frequency poker with money**		
Never	0 (0)	0 (0)
Sometimes	1 (4.3)	0 (0)
Often	4 (17.4)	1 (50)
Always	18 (78.3)	1 (50)
**PE 9_Frequency of use of tracking software**		
Never	9 (39.1)	1 (50)
Sometimes	1 (4.3)	0 (0)
Often	4 (17.4)	0 (0)
Always	9 (39.1)	1 (50)

### Questionnaires

The questionnaires are presented in order of administration.

**Eligibility questionnaire** is a 10-item questionnaire addressing OP gambling habits, age, time dedicated to other gambling activities, and work schedule.

**Socio-demographic questionnaire** is 15 items collecting data on marital and civil status, occupation, level of education, annual income, etc.

**Gambling Habits Questionnaire**. Inspired by the questionnaire by Lévesque et al. (47), 13 self-report items assessed the participant's gambling habits by collecting data on expenses related to gambling, time spent gambling, frequency, and gains/losses associated with poker and OP.

**Poker Experience** (PE, 24), a French translation, measure the level of experience of OP players. The PE is a self-report questionnaire consisting of nine items on a 4-point Likert-type scale measuring player's perception of their level of experience with poker (years of experience, frequency of play, books read, etc.). The original EP has good internal consistency (Cronbach's α = 0.70, 24).

**Problem Gambling Severity Index (PGSI)** is a subsection of the Canadian Problem Gambling Index (CPGI) and is used to measure the severity of problem gambling in the last 12 months. The scale consists of nine items rated on a 4-point Likert scale with answer options ranging from never to almost always. A score of 0 indicates non-problematic gambling, a score of 1–4 indicates low-risk gambling, a score of 5–7 refers to moderate-risk gambling, and a score of 8 or higher qualifies the gambling as problematic and possibly pathological (48). The PGSI items were asked twice, once for gambling in general (PGSI) and once for OP, producing a score specific for OP (PGSI-OP). The score of the PGSI-OP is interpreted in the same way as the score of the PGSI. This method has already been used (49).

**CPGI-Adverse Consequences on Individuals, Families, and Communities (CPGI - Consequences)** is a self-report 10-item questionnaire, measured on a 4-point Likert-type scale, that assesses the consequences of gambling in several areas of a person's life [interpersonal, marital, family, work, and community, (50)]. Items are modified to replace the terms “gambling” by online poker to solely address the consequences of this specific gambling activity.

**Sleep Diary** (51) is a daily nine item self-report questionnaire asking about: (a) the time at which the person attempts to fall asleep and the time at which the persons wakes-up, (b) time awake during the night, (c) perceived feelings of being rested (rated via a 5-point Likert scale), (d) the use of alcohol (yes or no), caffeinated beverages (yes or no) or drugs (stimulants, cannabis, hallucinogens or other) during the previous evening and (e) the partake in gambling activities other than OP during the previous day. The Sleep Diary used is a shortened and slightly modified version of Carney et al. (51). A question regarding drug use was added. The Sleep Diary also collected data such as sleep onset latency, total sleep time, and sleep efficiency. Sleep onset latency refers to the amount of time between turning off the lights with the intention to sleep and falling asleep. Total sleep time refers to the estimated time spent asleep, calculated from the time of attempted sleep to the time of awakening. Time spent awake during the night must also be deducted from the total sleep time. Sleep efficiency is defined as the proportion of time asleep out to the total time spent in bed.

**Online poker session schedules questionnaire** was created to survey participants about the start and end times of each OP session played the day before. This questionnaire ensured that the hours of each OP session were recorded even if hand histories were not provided by the participants.

**Online Poker Tilt Scale (OPTS)** is composed of 17 self-report items and is a validated measure to assess tilt episodes in OP players (27). This scale is scored on a 5-point Likert scale and is divided into two factors: (a) emotional and behavioral tilt (12 items) and (b) cognitive tilt (five items). The OPTS has good internal consistency (Cronbach's α > 0.80 for the total score and each subscale) and the average inter-item correlation is 0.46. This questionnaire also has good convergent validity, as it is significantly correlated with the number of tilt episodes experienced (past 3 months; *r* = 0.50; *p* < 0.001) and with the sub-types of gamblers found in the PGSI (PGSI; *r* = 0.77; *p* < 0.001).

### Software

**Hold'Em Manager 2** is an OP hand tracking software, by Max Value Software (https://www.holdemmanager.com). Hold'Em Manager 2 transforms the text files of the hand histories into summarized and detailed data of the hands and the OP sessions played. Among the summarized statistics, the net gains/losses and the number of hands played per session were used for this study.

### Procedure

Gamblers interested in participating in the study were contacted by the first author via telephone or Skype to verify their eligibility, to complete the verbal consent form and to complete the interview (socio-demographic questionnaire, gambling habits questionnaire, EP, PGSI, and CPGI-consequences). Players were then e-mailed information about completing the daily questionnaires as well as the procedure to activate the hand history tracking system. At the end of the interview, the researcher ensured that participants were able to activate the hand histories and, if necessary, assisted the participants. The completion of Sleep Diary, OPTS, and OP session schedules were carried out for 4 weeks on the secure web-based LimeSurvey platform. A daily e-mail was sent to the players as a reminder.

During the experimental period, participants completed the Sleep Diary at the beginning of each day. The OPTS and OP session schedule questionnaire were also completed if they had played OP the previous day. The player was asked to send their hand histories via e-mail after each week of data collection. Per each week of experimentation, the participants received $5 per day of participation in the form of a gift card (7 days x $5 = $35 gift card). The player was compensated if they completed the daily questionnaires, regardless of whether or not a session was played. The present study has a natural quasi-experimental design with control condition. The control condition is non-equivalent to the experimental condition and it was distinguished by session characteristics. This study has received ethical approval from the ethics committee of Université Laval, approval number 2017-338 A-3.

### Statistical Analyses

Statistical analyses were performed using the 23rd version of the software Statistical Package for the Social Sciences (SPSS). Descriptive statistics were performed on responses to socio-demographic questions, gambling habits, PE, the PGSI and OPTS for the duration of the experiment.

Mixed-design analyses of variance (ANOVAs) were conducted to test the hypotheses of the study. When the basic statistical assumptions were not met, the data were transformed (logarithmic, square root, rank, or normalized rank).

To achieve the main objective of exploring the effects of sleep deprivation on tilt symptoms and gambling behaviors, a mixed-design analysis of variance (ANOVA) was conducted for each dependent variable: total OPTS, emotional & behavioral tilt, cognitive tilt, net gains, or losses (in US dollars) and number of hands played. When a statistically significant group effect was observed, *post-hoc* ANOVAs were used to test for the presence of confounding variables related to alcohol, cannabis, stimulant, and hallucinogen use. Hand histories were not provided by four players in the sample. Another player provided only partial gambling session data, sometimes having played OP sessions for which it was not possible to obtain hand histories. As a result, self-reported session end times were used in the analyses on the self-reported dependent variables (OPTS and Sleep Diary variables) for these players. Players who did not provide hand histories were not included in the analyses of gambling behavior variables (net winnings and losses in US dollars and number of hands played). Hand history data was used to calculate sleep deprivation in the gambling behavior variable analyses for the player who provided only partial gambling information. To achieve the secondary objective of exploring the effect of evening OP sessions on sleep variables, a mixed-design ANOVA was conducted for each DV: time of attempted sleep, sleep onset latency, sleep efficiency, total sleep time, and feeling rested upon awakening in the morning. *Post-hoc* analyses were conducted using a mixed regression model to explore the association between tilt symptoms (emotional and behavioral tilt and cognitive tilt) and sleep dependent variables. For this purpose, the database was split according to whether an OP session was played the evening before or not (OP-Evening or NOP-Evening). The results for these analyses are presented this way.

### Sample

For this study, the sample does not consist of individual gamblers, but rather of data from gambling sessions. To achieve the primary objective of the present study, a total sample size of 588 gambling sessions were collected. Two states of wakefulness were compared: (a) sleep deprivation (SDpr), which is categorized as a session having ended at least 16 h since the person woke up and (b) a non-sleep-deprived (NSDpr) condition consisting of all other gambling sessions. The Sleep Diary provided information regarding the participants' wake-up time and information regarding the hour of the end of the gambling session was provided by the hand history feature of the tracking software. During the data collection, which lasted between 10 and 35 days (*M* = 27.19; *SD* = 6.39), the average gambling session ended 11.06 h (*SD* = 5.43) after waking up in the morning [0.17; 22.57], 80.1% (*n* = 479) of sessions were played while NSDpr and 19.9% were played while in SDpr (*n* = 119).

To achieve the secondary objective, a total sample of 897 observations was collected. The independent variable was operationalized as the presence or absence of an OP session before falling asleep. Two conditions were compared: (a) the presence of an OP session between 1 and 120 min before trying to fall asleep (OP-Evening) and (b) the absence of an OP session between 1 and 120 min before trying to fall asleep (NOP-Evening). In the NOP-Evening condition, participants could either have played no OP sessions that day or sessions could have been played more than 120 min before trying to fall asleep. Twenty-one percent (*n* = 190) of sessions were grouped in the OP-Evening condition and 78.8% (*n* = 707) were grouped in the NOP-Evening condition.

## Results

### Sleep Deprivation and Tilt Levels

As predicted, the mixed model ANOVA yielded a statistically significant difference on the OPTS Total with a higher score being observed in the SDpr condition in comparison with NSDpr condition (see Table 4). The final model also indicated that the OPTS Total score was significantly higher when the session was played while consuming alcohol [*F*_(1,488)_ = 5.19, *p* = 0.0023] (*M* = 1.95; *SD* = 0.14) vs. when no alcohol was consumed (*M* = 1.75; *SD* = 0.13) and when the session was categorized as SDpr [*F*_(1,485)_ = 5.16, *p* = 0.024] (*M* = 1.95; *SD* = 0.14) vs. when it was categorized as NSDpr (*M* = 1.75; *SD* = 0.13). There was no statistically significant difference in alcohol consumption between sessions in the SDpr condition (29.91%) and the NSDpr condition (32.21%).

**Table 4:** Degrees of freedom, means of NSDpr condition and SDpr condition, mixed model analysis of variance of symptoms of tilt according to total OPTS scores and OPTS subscales and gambling behaviors.

**Variables**	**df numerator**	**df denominator**	**Mean SDpr**	**Standard error**	**df**	**Mean NSDpr**	**Standard error**	**df**	***F***	***p***
OPTS_Total^a^	1	487	1.90	0.14	30.96	1.71	0.13	19.51	4.77	0.029
OPTS_Emotional & behavioral^a^	1	492	1.44	0.16	27.96	1.20	0.15	19.54	7.57	0.006
OPTS_Cognitive^a^	1	500	1.16	0.13	27.86	1.12	0.12	19.82	0.25	0.620
Net gains/losses (USD)^b^	1	528	−0.28	0.12	39.47	−0.18	0.09	12.53	5.91	0.015
Number of hands played^a^	1	559	5.08	0.25	23.65	4.73	0.23	16.48	6.64	0.010

a Data transformation = logarithmic transformed scores, ln(Var +1).

b Data transformation = standardized scores.

As hypothesized, the mixed model ANOVA also showed a statistically significant group effect on OPTS emotional and behavioral score, being higher in the SDpr condition in comparison to the NSDpr condition (see Table 4). The final model suggested that the OPTS emotional and behavioral score was significantly higher when the player had consumed alcohol [*F*_(1,497)_ = 5.89; *p* = 0.016] (*M* = 1.48; *SD* = 0.16) in comparison to when no alcohol was consumed (*M* = 1.26; *SD* = 0.15) and when the session was categorized as SDpr [*F*_(1,490)_ = 8.24; *p* = 0.004] (*M* = 1.49; *SD* = 0.16) vs. when it was categorized as NSDpr (*M* = 1.24; *SD* = 0.15).

Contrary to our hypothesis, the mixed model ANOVA did not reveal a statistically significant difference in OPTS cognitive score between the two conditions.

### Sleep Deprivation and Gambling Behaviors

As predicted, the mixed model ANOVA yielded a statistically significant group effect on the net gains/losses by the participants. Indeed, the mean net gains/losses amount was shown to be lower in the SDpr condition when compared to the NSDpr condition (see Table 4). *Post-hoc* analyses performed on alcohol, cannabis, stimulant, and hallucinogenic consumption did not reveal any statistically significant differences in the final model based on the conditions.

As hypothesized, the mixed model ANOVA also showed a statistically significant group effect on the number of hands played. The average total number of hands played was higher in the SDpr condition in comparison to the NSDpr condition (see Table 4). *Post-hoc* analyses performed on alcohol, cannabis, stimulant, and hallucinogenic consumption did not reveal any statistically significant differences in the final model based on the conditions.

### Online Poker Sessions Before Bedtime and Sleep

The following results refer to comparisons between the OP-Evening condition and the NOP-Evening condition (see Table 5). Contrary to our hypothesis, the mixed model ANOVA did not reveal a statistically significant group effect between conditions regarding the hour of attempted sleep, total sleep time, sleep efficiency, or in feeling rested in the morning. For sleep onset latency, the mixed model ANOVA revealed a statistically significant group effect. Indeed, sleep onset latency was shown to be longer in the NOP-Evening group contrary to our hypothesis.

**Table 5:** Mixed model analysis of variance on OP-Evening and NOP-Evening groups for sleep quality variables.

**Variables**	**df numerator**	**df denominator**	**Mean NOP-Evening**	**Standard error**	**df**	**Mean OP-Evening**	**Standard error**	**df**	***F***	***p***
Hour of attempted sleep^c^	1	761	435.03	37.17	28	415.69	35.66	24	1.98	0.16
Sleep onset latency^d^	1	762	358.83	33.24	31	397.04	31.15	24	6.26	0.013
Total sleep time^d^	1	769	377.51	27.63	38	394.94	24.30	23	0.99	0.32
Sleep efficiency^d^	1	754	379.99	30.42	33	386.58	28.05	24	0.18	0.67
Feeling rested in the morning	1	759	3.26	0.15	35	3.32	0.13	23	0.43	0.51

c Data transformation = ranked scores.

d Data transformation = standardized ranked scores.

### Tilt and Sleep

Five *post-hoc* analyses were conducted to better understand the effects of tilt on sleep quality measured by the hour at which the player attempted to go to sleep, sleep latency, total sleep time, sleep efficiency, and the reported feeling of being rested the following morning. These measures were compared based on the hour at which the last OP session was played (OP-Evening or NOP-Evening). As shown in Table 6, the first mixed regression analysis showed a significant positive association between time of attempted sleep and OPTS emotional and behavioral score for both OP-Evening and NOP-Evening sessions. The second mixed regression analysis yielded a significant negative association between sleep onset latency and OPTS emotional and behavioral score for OP-Evening sessions. Subsequently, a negative association between total sleep time and OPTS emotional and behavioral score was found in both conditions (OP-Evening and NOP-Evening. A negative association was also found between sleep efficiency and OPTS cognitive score for the NOP-Evening condition. Finally, the last analyses did not reveal any statistically significant association.

**Table 6:** *Post-hoc* Mixed regression analysis for sleep quality variables according to the time of the last online poker session between the two tilt factors.

**Sleep quality**	**Moment of the last online poker session**	**Predictors**	**dl numerator**	**dl denominator**	**Estimation**	***Standard error***	***F***	***p***
Time of attempted sleep^c^	OP-Evening	*OPTS_Emotional & Behavioral*	1	158	11.81	3.31	12.77	0.000
		*OPTS_Cognitive*	1	159	−2.09	6.02	0.12	0.730
	NOP-Evening	*OPTS_Emotional & Behavioral*	1	312	10.60	3.23	10.77	0.001
		*OPTS_Cognitive*	1	316	−5.29	6.03	0.77	0.380
Sleep latency^c^	OP-Evening	*OPTS_Emotional & Behavioral*	1	163	−8.86	4.31	4.23	0.041
		*OPTS_Cognitive*	1	164	8.14	7.80	1.09	0.300
	NOP-Evening	*OPTS_Emotional & Behavioral*	1	316	−2.62	3.72	0.49	0.480
		*OPTS_Cognitive*	1	319	−2.28	6.91	0.11	0.740
Total sleep time^c^	OP-Evening	*OPTS_Emotional & Behavioral*	1	168	−15.16	4.70	10.40	0.002
		*OPTS_Cognitive*	1	169	6.52	8.50	0.588	0.440
	NOP-Evening	*OPTS_Emotional & Behavioral*	1	318	−8.47	4.21	4.06	0.045
		*OPTS_Cognitive*	1	318	3.39	7.79	0.19	0.663
Sleep efficiency^c^	OP-Evening	*OPTS_Emotional & Behavioral*	1	168	2.81	4.62	0.369	0.540
		*OPTS_Cognitive*	1	168	−12.31	8.68	2.01	0.160
	NOP-Evening	*OPTS_Emotional & Behavioral*	1	317	6.81	4.15	2.69	0.100
		*OPTS_Cognitive*	1	309	−18.7	7.75	5.82	0.016
Feeling rested in the morning	OP-Evening	*OPTS_Emotional & Behavioral*	1	160	−0.006	0.025	0.057	0.810
		*OPTS_Cognitive*	1	159	0.028	0.046	0.374	0.540
	NOP-Evening	*OPTS_Emotional & Behavioral*	1	315	0.000	0.022	0.00	0.990
		*OPTS_Cognitive*	1	304	0.010	0.040	0.071	0.790

c Data transformation = ranked scores.

## Discussion

The purpose of this study was to explore the relationship between sleep and at-risk gambling behaviors. The first objective was to study tilt when OP was played in SDpr. As hypothesized, higher total tilt scores were observed when OP sessions were played in SDpr compared to those played while NSDpr. Total tilt scores were significantly higher when alcohol was consumed as well. When the two tilt factors are considered separately, higher emotional and behavioral tilt scores are observed if the sessions played are in SDpr, but no statistically significant difference was observed for cognitive tilt scores. Emotional and behavioral tilt is also higher when the player has consumed alcohol.

Emotional and behavioral tilt is characterized by negative emotions such as frustration, anger, a sense of loss of emotional control as well as by acting out during the OP sessions (e.g., “I play without thinking about the consequences”) or in actions surrounding OP session (e.g., “I throw things around or I attack my mouse”). In this study, higher emotional and behavioral tilt scores were observed during sessions where the player was in SDpr and when alcohol was consumed before or during the gambling session. There are few empirical studies exploring tilt in different contexts, but the results concerning emotional and behavioral tilt are consistent with the results of studies on emotional reactions, emotional regulation and acting out behaviors in SDpr.

Sleep is thought to play a role in the expression of emotions. However, SDpr may contribute to alterations in this function (22, 39). Among the studies identified by Watling et al. (22), only Zohar et al.'s (41) study was conducted in a natural setting. Conducted among 78 physicians on duty during the first 2 years of their residency, this study examined the relationship between the emotions reported following various professional situations and sleep. In the context of SDpr (measured using a numerical ActiGraph^1^), residents reported more negative emotions when experiencing unexpected or disruptive events and fewer positive emotions following successful outcomes compared to the resting state. Although medical residents and OP players may differ in several ways, the study by Zohar et al. (41) illustrates that altered sleep patterns can have a negative impact on the emotional experience in an ecological context where participants have a level of experience and knowledge of the context in which the study takes place. The SDpr sessions in our study could thus be associated with an increase in frustration, anger or other emotions when an unexpected or disruptive event occurs, thereby promoting tilt symptoms. As reported by poker players (28), these events may occur during the OP session (e.g., bullying by another player, losing when the odds are in favor of winning, following a bad sequence of play) and be either internal (e.g., inattention) or external (e.g., conflict during the day) in nature.

Sleep is also thought to play a role in emotional regulation (22). However, as for the expression of emotions, SDpr may also impair a person's ability to regulate emotions (40, 42). Impaired emotional regulation is characterized by difficulty in observing, assessing, and modulating emotions to achieve goal-directed behaviors (22, 52). Based on the results of Mauss and Talbot's studies (40, 42), the higher level of emotional and behavioral tilt observed in our study during sleep-deprived OP sessions could be explained not only by a different rapport to emotions, but also by an impairment in the ability to regulate those emotions. Without being able to adequately mentalize their internal states, it would be more difficult for the sleep-deprived OP player to take a step back from the situation and adopt regulatory strategies to reduce the intensity of emotions. Therefore, a greater propensity to act out may be observed as measured by some OPTS items (e.g., “I click faster and hit my keyboard harder,” “I shout and insult other people,” “I play without thinking about the consequences”). These behaviors then correspond to the externalization of emotions that could not be adequately regulated.

While gambling sessions played while sleep-deprived may lead to more emotional and behavioral tilt, this effect is not observed for cognitive tilt (e.g., “I am less focused; I take more risks; my decisions are no longer rational; I don't feel like myself; it's like I have no control over the game”). This result contradicts the original hypothesis which was based on several research findings suggesting that SDpr has an effect on cognitive abilities, decision-making capacities. and risk-taking behaviors of participants in laboratory studies (9, 32, 34, 38). It is possible that sessions played in SDpr simply did not promote cognitive tilt episodes for our sample. That is, gamblers did not experience changes in their level of concentration, their risk-taking propensity, their decision-making abilities, their feelings of dissociation or loss of control over gambling when they were sleep-deprived. This interpretation could be supported by the level of experience of the players in our sample as well as using tracking software during the sessions. In fact, more than 75% of the participants in our study have been playing poker for more than 5 years and almost half of the participants' sample perceived they had an excellent knowledge of poker statistics and probability. More than 60% of the players in our sample used a tracking strategy during their sessions. In addition, all the players reported having already played OP while sleep-deprived in the past. Given their level of experience and the use of tracking strategies for the majority of the sessions, it is possible that these players were able, to some extent, to maintain their gambling strategy and limit the loss of control even while in a state of SDpr and potentially in a episode of emotional and behavioral tilt. This would be consistent with the results of Morgan's (25) study where experienced gamblers did not experience an increase in risk-taking behaviors as a result of situations that induced negative emotions. Despite the lack of significant results between groups for cognitive tilt, gambling sessions played in SDpr did have unfavorable financial outcomes compared to sessions played at rest.

The hypothesis that greater net losses will be observed in sessions played while in SDpr compared to sessions played while NSDpr was confirmed. This result suggests that playing while sleep-deprived may lead to unfavorable financial outcomes. This finding is most likely explained by the adverse effects that SDpr has on decision-making ability, risk-taking (9, 32, 34, 38) and emotional regulation (40, 42). Concretely, this variation in net losses could be explained by a greater diversity in gambling styles (e.g., the aggressiveness of the player, risk-taking, etc.) leading to more losses when the player is sleep-deprived. This would be consistent with the results found by Womack et al. (34) and Demos et al. (37) who noted that SDpr promotes increased risk-taking and impulsivity. However, this interpretation is not supported in our study as no difference was detected between groups with respect to the item on risk-taking in the cognitive tilt factor. It is, however, important to note that cognitive tilt does not specifically measure risk-taking, as the OPTS is not necessarily sensitive enough to detect a variation in participant's risk-taking behaviors. An alternative interpretation can be found when considering the findings related to emotional and behavioral tilt. It is possible that results pertaining to financial outcomes revealed in this study may partially be explained by the higher level of emotional and behavioral tilt symptoms in the SDpr group. Indeed, tilt is associated with a loss of control over gambling behaviors and more monetary losses (28, 31). From this perspective, emotional and behavioral tilt would better explain financial outcomes then sleep deprivation state. It would be beneficial to test these two explanatory hypotheses in future studies.

It was also observed that more hands were played in SDpr sessions. For this result, it is difficult to offer an explanation based on a potential loss of control of gambling behaviors when sessions are played in SDpr as the data from this study was collected from sessions played in both cash games and tournaments. Thus, more hands do not indicate the same phenomenon for both conditions. A gambler who plays more cash games is more likely to lose because of the possibility to put more money back into the bankroll. However, more hands played in a tournament is an indication that the player is getting further in the competition: there is no possibility to add extra money into the bankroll, however there is a better chance of recovering expenses from the buy-in and even making a profit.

Ultimately, gambling sessions played in SDpr indicate that gambling while sleep-deprived is a risky practice for the players in our sample. In fact, players who often gamble while sleep-deprived may incur more losses and financial debt. Similarly, players who gamble a greater number of hands while sleep-deprived may experience negative impacts in regard to their daytime occupations, their relationships or work activities. In fact, almost a third of the gamblers in our sample reported that OP may have caused complications in their partner's life. However, these hypotheses should be tested in longitudinal studies.

Conversely, the tilt episode itself can adversely affect the players' sleep. In our sample, emotional and behavioral tilt was associated with participants having a later bedtime and less total sleep hours regardless of when the sessions were played. This finding implies that players experiencing tilt symptoms go to bed later, irrespective of the time the session was played, suggesting that the effects of tilt may extend over several hours. This result provides a nuance to findings observed in the qualitative study by Moreau et al. (28), in which players describe tilt as a transitory phenomenon that passes when the player leaves the gambling table. It is possible that more time is needed to relax before going to bed after a tilt episode is experienced. Following episodes of tilt, players report a tendency to ruminate and experience a range of emotions such as disappointment, anxiety (26), guilt, sadness and disgust (28). A great deal of emotional regulation may be required to prevent these emotions from impairing sleep quality (22). The association between the emotional and behavioral factor of tilt and total sleep time is consistent with these findings. Players in our sample experiencing emotional and behavioral tilt symptoms go to bed later and therefore sleep fewer hours. Further studies are necessary to better understand the effects of tilt on the time of attempted sleep and total sleep time.

Emotional and behavioral tilt is also associated with a shorter sleep onset latency when the session is played 2 h before bedtime. This result can be interpreted in terms of participant's later bedtime, a variable that is also influenced by tilt. Gamblers experiencing emotional and behavioral tilt episodes may be inclined to go to bed later, leading to greater feelings of exhaustion and therefore a shorter sleep onset latency. However, further research is needed to confirm this interpretation. Finally, our results show that cognitive tilt is associated with a decrease in sleep efficiency the night following an OP session when this session is played more than 2 h before bedtime. As highlighted in the results of Browne's (26) qualitative study as well as outlined by certain OPTS items, cognitive tilt may cause the player to ruminate about the consequences of poor decision making. Based on this interpretation of the results, a longer period between the end of the session and bedtime could lead to an exacerbation in the player's rumination. This can, in turn, have an effect of sleep quality as rumination is amongst the symptoms that impair sleep and contribute to long-term sleep problems (43). Curiously, cognitive tilt is only associated with reduced sleep efficiency and not with other sleep quality variables. Future studies exploring rumination in the context of a tilt episode and its effects on sleep could further contribute to our understanding.

The secondary objective of this study was to explore whether or not OP sessions played near bedtime has an effect on sleep quality. More specifically, it was hypothesized that OP sessions played in OP-Evening condition would result in a later bedtime, increased sleep latency, decreased sleep efficiency, shorter total sleep time, and feeling less rested the following morning compared to NOP-Evening condition. These hypotheses were all refuted and, in fact, a shorter sleep onset latency was observed when sessions were played 2 h before bedtime. It was expected that gambling shortly before bedtime would have adverse effects on sleep, either by interfering with sleep (6), due to the emotional stimulation that playing may provide (46) or by increasing rumination before bedtime (43). However, it appears that an evening OP session does not yield any of these adverse effects to such an extent to affect sleep quality in participants.

Another surprising result was the observation of a shorter sleep onset latency when playing OP at night. This finding was unexpected and raises the question of whether OP can help players go to sleep. On one side, shorter sleep onset latency observed after an evening session could indicate that OP has a role in regulating players' emotions before bedtime, thus the shorter sleep latency after an evening OP session. This comprehension is supported by Wood et al. (15) results, in which problem gambling was predicted by playing to escape problems. In fact, almost two-thirds (60.9%) of our sample are low-risk gamblers and 30.4% are either moderate or possibly pathological gamblers (PGSI-OP). In this context, OP may be beneficial for players' sleep in the short term but may have adverse consequences if the player needs to play in order to have a good night's sleep. On the other hand, it is also possible that OP is part of an evening routine for the players in our sample. Referring to Morin's (53) recommendations for the treatment of insomnia, a consistent sleep routine is an integrative part of an overall sleep hygiene. It is possible that the players in our sample found OP to be a relaxing activity associated with pleasure which may explain the shorter sleep onset latency observed. However, our study did not explore the motivations to play or other aspects in the gambler's nighttime routine other than OP and therefore these interpretations of the results must be addressed by future studies.

### Strengths and Limitations

The results of this study must be considered in light of certain strengths and limitations. Firstly, the research protocol used allowed us to collect objective OP data as well as subjective data on tilt episodes and daily sleep variables. The daily questionnaires allowed us to observe changes in the key study variables over 24-h periods, ultimately allowing us to gain a better understanding on how these variations may interact with each other. This close follow-up also made an ecological study design possible for the key variables. The use of mixed-analyses statistics permitted comparisons of gambling sessions based on the time of day they were played rather than separating gamblers into groups, ultimately providing statistical control for the intra-group variance. This ensures that sessions played by a subgroup of participants do not, in themselves, explain the observed differences in conditions. It also provided access to a large pool of gambling sessions providing good statistical power. However, due to our research protocol, data regarding key variables (OPTS and sleep quality) could be collected over a 24-h period. Yet, one or several OP sessions could be played in the same 24-h period by participants, making it impossible to discriminate data between groups (SDpr vs. NSDpr), which may have negatively affected the statistical power of our analysis. Moreover, as daily data collection required a great deal of engagement and discipline from the participants, many daily questionnaires were left incomplete or empty. As a result, some gambling sessions could not be associated with the dependent variables, resulting in a loss of data. A similar study with more objective data such as the use of a digital ActiGraph watch would make it possible to offset this limitation.

Furthermore, the eligibility criteria for this study solely included OP players who occasionally played while in SDpr, defined as playing 16 h since awakening. Eligibility criteria also favored regular and more experienced players; thus, our sample included a high rate of problem and probable pathological gamblers according to the PGSI. It was not possible to observe how gambling problems interacted with the variables under study. Thus, the results are not generalizable to all OP gamblers, but rather to regular OP gamblers who gamble frequently late at night or in the evening. Finally, our cross-sectional design does not allow causality to emerge, however our protocol allows us to observe a temporal link between our main variables.

### Clinical Implications

The results of this study have clinical implications for public health and health professionals. Poker players should be informed that their sleeping habits have repercussions on tilt symptoms and loss of control while gambling, especially if they use alcohol. Playing poker while sleep deprived could have lingering effects on various spheres of their lives. Working on changing harmful sleep habits should be one of the goals of therapy for poker players who gamble at night.

## Conclusion

In summary, the objective of this study was to explore the relationship between sleep problems and risky gambling behaviors of OP players' gambling sessions based on the time of day at which they were played. The results from our study suggest that higher levels of emotional and behavioral tilt are present for sessions played sleep-deprived (SDpr condition) compared to when the player was well-rested (NSDpr condition). Alcohol consumption was also shown to have an impact on the level of emotional and behavioral tilt. No cognitive tilt symptoms differences were observed between SDpr and NSDpr conditions. However, larger number of hands and more losses/sessions were observed in the SDpr condition. This relationship was not affected by alcohol or substance use. In addition, there was no significant relationship found between sessions played 2 h before bedtime and sleep quality. Nevertheless, participants reported a shorter sleep latency when sessions were played 2 h before bedtime. Although our results suggest that OP has little impact on sleep, sleep does seem to be affected when tilt symptoms are reported. Our findings show that emotional and behavioral tilt is associated with later bedtime, decreased total sleep time and shorter sleep latency. Also, cognitive tilt is associated with decreased sleep efficiency when gambling sessions have not taken place 2 h before bedtime. More studies are needed to better understand the association between gambling behaviors and sleep patterns. To shed further light on our findings, future studies could explore the motives for late night OP playing. Future studies are also needed to explore what happens between a gambler's tilt episode and the time they go to sleep. Similarly, the inclusion of objective data on gambling and sleep patterns, via the use of a numerical ActiGraph for example, in future studies would provide further insight and enrich the interpretation of the results.

## Data Availability Statement

The raw data supporting the conclusions of this article will be made available by the authors, without undue reservation.

## Ethics Statement

The studies involving human participants were reviewed and approved by Comités d'éthique de la recherche avec des êtres humains de l'Université Laval (CERUL). The patients/participants provided their written informed consent to participate in this study.

## Author Contributions

This study is part of AH doctoral dissertation in psychology at Université Laval. He was co-supervised by IG and CB. AH led all parts of the study and the redaction process. IG had the original idea for the study. IG and CB supervised every step of the process, they revised every document, and provided their respective expertise in gambling and sleep research, they revised every document, and provided their respective expertise in gambling and sleep research. CJ research professional at the Center québécois d'excellence pour la prévention et le traitement du jeu also provided his expertise in gambling and helped a lot to increase the methodology quality. He also revised the article mostly toward the end of the process. AM provided her expertise regarding the online poker aspect of the article and the study. Her insights helped to reach online poker players. She also revised the document. All authors contributed to the article and approved the submitted version.

## Conflict of Interest

The authors declare that the research was conducted in the absence of any commercial or financial relationships that could be construed as a potential conflict of interest.

## Footnotes

^1^Instrument usually worn on the wrist that measures sleep/wake cycles via the participants' body movements.

## Abbreviations

CPGI: Canadian Problem Gambling Index
CPGI – Consequences: CPGI-Adverse Consequences on Individuals, Families, and Communities
NOP-Evening: No online poker session played within 2 h before trying to sleep condition
NSDpr: Online poker session is played while not sleep-deprived condition
OP: Online poker
OP-Evening: Online poker session played within 2 h before trying to sleep condition
OPTS: Online Poker Tilt Scale
PE: Poker Experience
PGSI: Problem Gambling Severity Index
PGSI-OP: Problem Gambling Severity Index specific to online poker
SDpr: Online poker session is played while sleep-deprived condition.
